# Economical Eating

**Published:** 1857-10

**Authors:** 


### ECONOMICAL EATING.

As a dog grows faster and fatter on a diet of two-fifths bread and three-fifths fish, than when fed on bread altogether, it is concluded that fish is more nutritive than bread, as to men as well as to dogs.

When the best beef steak is selling at twenty cents a pound, the butchers are glad to sell the "rein" piece at 8 or 10 cents a pound. It has no bone nor fat. Three pounds of this for twenty-five cents will make soup enough for a family of eight or ten persons two days, besides the meat for one dinner; it is a hundred per cent. cheaper than the purchase of a knee joint, at forty cents, for soup.

Of all the parts of corned beef, that is the most nutritious and cheapest which is called the round, which has neither bone nor gristle, nor waste fat worth naming.

Both in the purchase of meat and fish, persons are generally falsely economical in choosing an article with bone in it, at two, or three, or more cents a pound less than a piece which has none.

We purchase porgies, blue-fish, flounders, and the like, at six or eight cents a pound, instead of halibut, at twelve cents. But the halibut is cheapest, and also the safest for a family where there are children, as it has none but the back bone; with that exception, it has solid flesh, whereas, in purchasing the smaller fishes named, they are weighed out with heads, entrails, fins, bones, and all.

The halibut is caught on the banks of Newfoundland, weighs from one hundred to six hundred pounds, and is widely prized. It is a flat fish, like the flounder, swims at the bottom of the sea, has both eyes on one side of the head, and is very voracious; will swallow a sounding lead in a moment.

Immense quantities of knotty apples are brought to the New York market. They are dear at any price, beyond the cost of carriage to market, both from their great waste and unhealthfulness. A clear apple is a hundred per cent cheaper, at double the price of the same sized knotty ones, which we see here every day.

